# First study of heavy metals analysis in hair and oxidative status of European Otters (*Lutra lutra*) from Southwestern Europe

**DOI:** 10.1007/s10646-025-02911-x

**Published:** 2025-07-01

**Authors:** Javier García-Muñoz, David Fernández Casado, Ángel Portillo-Moreno, María del Prado Míguez-Santiyán, Francisco Soler Rodríguez, Ana López-Beceiro, Luis Eusebio Fidalgo, Salomé Martínez-Morcillo, Marcos Pérez-López

**Affiliations:** 1 Toxicology Area, Faculty of Veterinary Medicine (Universidad de Extremadura), Caceres, Spain; 2 Department of Veterinary Clinical Sciences, Faculty of Veterinary Medicine (Universidad de Santiago de Compostela), Lugo, Spain

**Keywords:** Biomonitoring, Biomarkers, Non-invasive sample, Inorganic elements, Otter

## Abstract

Heavy metal(loid)s are continuously released into semi-aquatic ecosystems. This poses a potential threat to wildlife, such as the European otter (*Lutra lutra*) whose population has been gradually declining. Non-invasive sampling (e.g., hair samples) offers a valuable approach to assess pollutant exposure in otters without harm. In this study, we quantified As, Cd, Hg, Pb, and Zn concentrations in the hair, liver, and kidney of European otters from NW Spain, with the aim of validating the effectiveness of hair as a non-invasive biomonitoring tool by comparing it with internal organ concentrations. The correlation between metal concentrations, age, sex, and habitat, as well as a battery of oxidative stress biomarkers (catalase, glutathione reductase, glutathione S-transferase and malondialdehyde production), were also assessed. Tissues from 28 otters were analyzed and metal concentrations were quantified by inductively coupled plasma mass spectrometry. Oxidative stress biomarkers, including antioxidant enzymes and lipid peroxidation, were determined in the liver and kidney by spectrophotometric methods. Significantly higher Hg concentrations were found in otter hair compared to liver and kidney, particularly in areas with local pollution sources. The rest of the heavy metal(loid)s were recorded at lower concentrations. Positive correlations were observed between Hg and As levels in hair and the liver and kidney. Statistically significant differences in Hg, Cd and Pb concentrations were found between adults and juveniles, as well as between males and females from coastal areas. Moreover, the correlations between heavy metal(loid)s and oxidative stress biomarkers reflected the physiological responses of individuals exposed to these pollutants. The present study is the first to quantify hair metal levels and oxidative status in the European otter from SW Europe. According to our findings, hair demonstrated its suitability as a non-invasive biomonitoring tool for assessing internal Hg and As concentrations, and could be considered in future long-term wildlife biomonitoring programs.

## Introduction

Aquatic environments, including freshwater and coastal ecosystems, constitute vital reservoirs of biodiversity but are increasingly threatened by toxic pollutants released by human activities. This environmental pollution contributes significantly to the degradation of these habitats, making them some of the most affected ecosystems worldwide (Saidon et al. 2024). Pollutants come from industrial processes, mining activity, urban runoff, and agriculture and represent a persistent and long-lasting hazard (Tchounwou et al. 2012; Gautam et al. 2014). Thus, intricate habitats are increasingly disturbed, disrupting the balance between ecosystem compartments.

Heavy metal(loid)s constitute a significant risk that profoundly impacts the delicate balance of semi-aquatic ecosystems (Saidon et al. 2024). Heavy metal(loid)s are naturally occurring inorganic elements with atomic numbers over 20 and an elemental density greater than 5 g cm^−3^ (Ali and Khan, 2018). Due to their physicochemical properties, these elements tend to be highly persistent in the environment and are toxic even at low concentrations. They are thus bioavailable to multiple organisms and bioaccumulate at different trophic levels (Ali et al. 2019). Heavy metal(loid)s have been classified as the most hazardous substances by the United States Agency for Toxic Substances and Disease Registry (ATSDR, 2022) with the following rank: arsenic (1), lead (2), mercury (3), and cadmium (7). These metal(loid)s do not perform any biological function and several studies have shown detrimental effects in living beings (Kalisińska, 2019b). The presence of these pollutants in aquatic environments has a domino effect with ecological repercussions that affects the overall ecosystem health.

Arsenic (As) is a metalloid widely used in many human activities, such as pesticide production and smelting of metals or fossil fuels; it is commonly found in water bodies (Alonso et al. 2014). Its toxicity relies on chemical speciation as inorganic forms tend to show higher toxicity (As^3^ and As^5^) (Osuna-Martínez et al. 2021). Despite the natural occurrence of lead (Pb) in the environment, its concentration has been increasing through human activities. This element is incorporated into water bodies and remains bioavailable as Pb^2+^, which has a higher mobility than the non-oxidized form (Lee et al. 2022). Mercury (Hg) poses a potential risk to aquatic ecosystems because of its ubiquity (Shore et al. 2011). Once mercury enters the water, microorganisms in the sediment methylate it into the organic form (Met-Hg), which is highly toxic and has a long biological half-life (Lehnherr, 2014; Kalisińska et al. 2019). Met-Hg can be absorbed by organisms and, under certain conditions, may bioaccumulate and biomagnify through trophic webs (Chételat et al. 2020). Cadmium (Cd) is mainly deposited on sediments via solid particles and is subsequently assimilated by the organisms (Saidon et al. 2024). In contrast to heavy metal(loid)s, other inorganic elements, such as zinc (Zn), have an essential role in the organism. Zn is involved in the synthesis of structural proteins or enzymes, as well as in metabolic reactions. Although Zn is relatively non-toxic to mammals, its toxicity depends on the concentration and duration of the exposure. Both the deficiency and abundance of Zn can lead to deleterious effects and its concentration reflects the health status of an animal (Kosik-Bogacka and Łanocha-Arendarczyk, 2019).

Biomonitoring studies are indispensable for assessing environmental quality and contaminant impact in ecosystems. Monitoring wildlife animals provides relevant information on natural conservation and potential human exposure to pollutants (Kalisińska, 2019a). Because the presence of many pollutants in the air, water, and soil poses significant risks to biodiversity and public health, the concept of “One health” is relevant to ecotoxicological studies because it emphasizes the relationships between human, animal, and environmental health. It is important to understand that this concept is focused on preventing and mitigating the effects of environmental pollution while ensuring global welfare (CDC, 2024).

The European otter (*Lutra lutra*) is a native mesocarnivore widely distributed in the Eurasian region and has a series of properties that make it a suitable biomonitoring model. Because this mustelid is an apex predator in semi-aquatic ecosystems, it has been commonly used as a sentinel species for risk assessment (Kalisińska et al. 2021; Baos et al. 2022; Regnery et al. 2024). The presence of critical levels of xenobiotics in semi-aquatic ecosystems, resulting in habitat alteration, has exerted significant pressure on otters, leading to a population decline to near-threatened status, according to the International Union for Conservation of Nature (IUCN) (Loy et al. 2022). In addition, this population regression has also been observed in Iberian otters, which are normally associated with regions with high levels of rainfall (Delibes et al. 2009; Ruiz-Olmo, 2014). Likewise, in the Iberian Peninsula as well as the rest of the European countries, several studies have identified high levels of environmental pollutants - particularly heavy metals and persistent organic compounds such as organochlorine pesticides, polychlorinated biphenyls (PCBs), or polycyclic aromatic hydrocarbons (PAHs) - as key contributors to habitat degradation affecting otter populations (López-Martín and Ruiz-Olmo, 1996; Mateo et al. 2012; Esposito et al. 2020; Dibbern et al. 2021).

Invasive sampling methods pose both ethical and ecological challenges (Millán et al. 2008; Lentini et al. 2017; García-Muñoz et al. 2023a). Non-invasive techniques offer a transformative approach, providing a nuanced understanding of contaminant dynamics while minimizing harm or stress. Their uses enable continuous and ethical monitoring that preserves the integrity of ecosystems, which helps to assess the exposure of wildlife - especially elusive or protected species such as the river otter - to contaminants and promotes the conservation of biodiversity (Hernandez-Moreno et al. 2013; Jota Baptista et al. 2022a; Valverde et al. 2024). Among non-invasive samples, hair is considered a relevant matrix for measuring metal(loid) exposure, as it reflects long-term accumulation and can provide information on an individual’s exposure history and environmental interactions (García-Muñoz et al. 2023b). Unlike Hg, the literature on the quantification of other metal(loid)s in European otter hair is scarce and has mainly focused on terrestrial wild mammals (McLean et al. 2009; Sánchez et al. 2022). Few studies have measured heavy metals in non-invasive otter samples from the Iberian Peninsula and the rest of the Mediterranean region. Most of them have used feces, while hair samples have not been addressed yet (Rodríguez-Estival et al. 2020; Baos et al. 2022).

Experimental studies in mustelids have found that hair metal(loid) concentrations have a strong association with the animals’ diet (Aulerich et al. 1974; Wang et al. 2014; Pitoňáková, 2023). Even though feces are widely available, easy to collect, and reflect the recent diet, they do not provide a long-term measure of metal(loid) exposure because they mainly contain unabsorbed material. Moreover, feces are less stable, as they are more susceptible to degradation or external contamination (Jota Baptista et al. 2022a). Interpreting fecal metal levels is challenging due to the variability in diet, ingestion, absorption, and excretion between organisms. According to the review by Jota Baptista et al. (2022b), only one study has reported inorganic elements concentrations in otters from the Iberian Peninsula, while focusing on invasive samples including liver and muscle (Hernández et al. 1985). It is thus clear that there is a lack of biomonitoring studies using European otters as a sentinel species for monitoring environmental pollution caused by heavy metal(loid)s in semi-aquatic ecosystems in SW Europe.

Exposure to hazardous pollutants, such as heavy metals, often induces the production reactive oxygen species (ROS) (Kalisińska, 2019b). An imbalance between ROS overproduction and antioxidant defenses can lead to increased oxidative stress, resulting in oxidative damage to membranes, lipid peroxidation, and the development of many diseases (Roméo et al. 2013). A ROS response can be detected using biomarkers, which are early and sensitive indicators of the degradation of an organism’s health (Isaksson, 2010). Previous biomonitoring studies have primarily focused on assessing various oxidative stress biomarkers in wild mammals, particularly in small and large species (Reglero et al. 2009; Quina et al. 2023). Nevertheless, no literature is available on the oxidative status and the biochemical effects of heavy metal in the European otter. The aims of the present study were (1) to investigate the current heavy metal(loid) burden (As, Cd, Hg, Pb, and Zn) in hair, liver, and kidney of the European otters from NW Spain; (2) to correlate the levels of metal(loid)s in liver, kidney, and hair to establish as a non-invasive sampling approach; (3) to assess bioaccumulation trends that take into account intrinsic (age and sex) and extrinsic factors (habitat); and (4) to apply a battery of oxidative stress biomarkers, including antioxidant enzymes (catalase, CAT, glutathione reductase, GR, and glutathione S-transferase, GST) and lipid peroxidation products (malondialdehyde, MDA) to obtain baseline data on the early responses of otters exposed to these pollutants.

The hypothesis of this study is that elevated levels of heavy metals in aquatic ecosystems may be associated with oxidative stress in otters. Given the correlations described in previous research between metal(loid) concentrations in internal organs and hair, hair samples are expected to reflect internal exposure, supporting the use of this non-invasive matrix as a reliable indicator of contaminant burden.

## Material and methods

### Sample collection

**Fig. 1:**
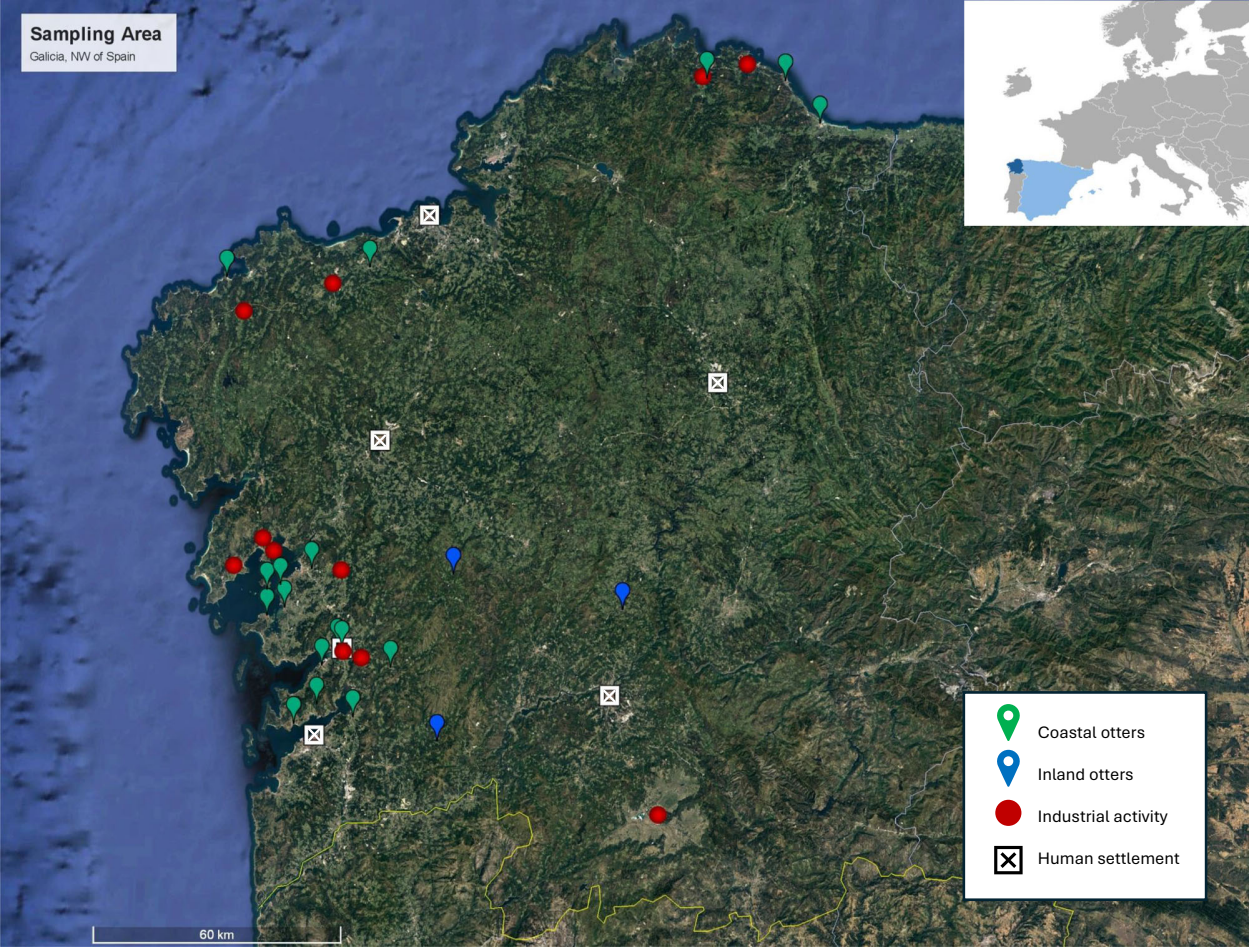
Distribution of the sampling areas for European otters in Galicia, Spain

European otters (*n* = 28) were collected during the period 2020–2022 in Galicia (NW Spain) (Fig. 1). The collected specimens were found death because of natural causes (i.e., infectious diseases) or road traffic accidents. In all cases, these animals were referred to the wildlife recovery centers in the region. Only animals that had been at the center for less than 5 days before dying were used for this study. Ethical approval was not required for this study, according to Directive 2010/63/EU of the European Parliament and of the Council (September 22, 2010) on the protection of animals used for scientific purposes educational activities. These regulations specify that approval is not required for studies on tissues or organs obtained from animals killed by another entity. Dead specimens were frozen and stored at – 80 °C until the necropsy was performed. Body mass ranged between 1.11 and 7.07 g. The animals were 14 males and 14 females. The animals’ age was estimated from the dental development, and the degree of sexual maturity (21 adult and 7 young), as previously established for different wildlife species (Pérez-López et al. 2016). Otters were classified based on their capture location according to the dispersal range proposed by Ruiz-Olmo (2014) (0.8 to 14.2 km). As a result, 25 otters were categorized as coastal and three as continental specimens.

Internal organ samples were taken from each animal, placed in individual plastic bags, and stored until metal(loid) analysis could be performed. To avoid metal contamination and losses, plastic scalpels and surgical tools were cleaned or replaced for sampling each animal. The working surface was also cleaned after each necropsy. Samples were handled avoiding contact with other metallic surfaces (Hernández-Moreno et al. 2013).

### Metal determination

To analyze heavy metal concentrations in hair samples, the methodology described by Hernández-Moreno et al. (2013) was used. Hair samples were first washed to remove all surface contamination. Hair was washed with tap water, distilled water, and acetone using a mixer (IKA^®^ TRAYSTER digital) in a 5-min cycle. The process was repeated three times. About 3–4 g of liver and kidney samples and 1–2 g of hair were dried to a constant weight in an oven for 72 h at 65 °C. After drying, the tissues were transferred into sealed 15 mL conical centrifuge tubes (Falcon®).

Heavy metals were analyzed at the Elemental and Molecular Laboratory of the Research Support Service (SAIUEX, accredited by ISO 9001:2008; University of Extremadura). For heavy metal quantification, 0.3–0.5 g of liver, kidney, and hair were used. For sample digestion, 3 mL HNO_3_ (69%) and 1 mL H_2_O_2_ (3:1 ratio) were added using sealed Teflon PTFE flasks. The digestion was carried out in a microwave automatic digester (Milestone Ultrawave 1) at 200 °C and 120 bar for 15 min. The resulting solutions were diluted to 25 mL with milliQ water. As, Cd, Hg, Pb, and Zn concentrations were analyzed by inductively coupled plasma mass spectrometry (ICP-MS) with an autosampler (Agilent Technologies Model 7900). The limit of detection for all metals was 0.003 mg kg^−1^, and the limit of quantification 0.005 mg kg^−1^. Each sample batch included blank and initial calibration standards, using a certified lyophilized bovine liver sample (BCR®, ref 185 R, Community Bureau of Reference, EU) as reference. The concentrations of As, Cd, Hg, Pb, and Zn were reported in mg kg^−1^ on a dry weight (dw) basis, because of the recognized reliability and consistency of dry values compared to wet weight values (Adrian and Stevens, 1979). For easier comparison with literature reporting concentrations in wet weight (ww), we adjusted the concentration by calculating the percentage moisture content of each sample during the drying process.

### Oxidative stress biomarkers in liver and kidney

Enzymatic (catalase, CAT, glutathione reductase, GR, and glutathione-S-transferase, GST), and non-enzymatic oxidative stress biomarkers (malondialdehyde, MDA) were analyzed using a spectrophotometer (BioTek FL600). About 0.5 g of liver and kidney were weighed, placed in glass tubes, and kept on ice for slow thawing. Five mL of phosphate buffer (0.1 M and 7.4 pH) was added to each sample and mixed using a homogenizer 20HS rod (PCU Kinematica). All samples were centrifuged at 3500 rpm for 5 min (Centronic S-577) to obtain the supernatant fraction. For non-enzymatic responses, MDA was estimated as thiobarbituric acid-reactive substances (TBA). Part of the supernatant (50 µL) was precipitated by adding perchloric acid at 70% and centrifuging (4000 rpm, 15 min, 4 °C in a DIGICEN 21 R centrifuge). A mixture of TBA and trichloroacetic acid was added and samples were incubated for 1 h at 80 °C. The colorimetric reaction was measured at 532 nm according to the method described by Recknagel et al. (1982). MDA was expressed as nmol mg^−1^ protein. For enzymatic responses, and to measure oxidative stress biomarkers, the supernatants were centrifugated at 12000 rpm for 20 min at 4 °C. CAT activity was analyzed using the methodology described by Clairborne (1985), based on the reaction of H_2_O_2_ to decompose into H_2_O and O_2_, measuring absorbance at 240 nm, and expressed as one enzyme milliunit (mU) which is the amount of CAT capable of transforming 1.0 nmol of H_2_O_2_ in 1 min. The molar extinction coefficient used was ε = 40 M^−1^ cm^−1^. For assessing GR activity, a modification of the approach outlined by Cribb et al. (1989) was used. A volume of 7.5 µL of the sample was mixed with nicothiamide adenine dinucleotide phosphate, oxidized glutathione, and diethylene-triamino-pentaacetic acid, previously diluted with buffer (0.05 M and 7.0 pH). Enzyme activity was measured at 340 nm using the extinction coefficient ε = 6220 M^−1^ cm^−1^ and expressed as nmol min^−1^ mg^−1^ protein (mU mg^−1^ protein). GST activity was determined following the procedure outlined by Habig et al. (1974), using 1-cloro-2,4-dinitrobenzene as substrate in combination with reduced glutathione (GSH), the change in absorbance at 340 nm was measured using the extinction coefficient ε = 9.6 × 10^3^ M^−1^cm^−1^ and expressed as mU mg^−1^ protein. All biomarkers were assessed relative to protein concentrations, which were analyzed using the Bradford (1976) method. Oxidative stress biomarker levels were expressed relative to the protein content (in mg) in the homogenates.

### Statistical analysis

All data were analyzed using GraphPad Prism 9.0.2 (GraphPad Software Inc., La Jolla, CA, USA). Data were expressed as mean ± standard error of the mean (SEM), standard deviation (SD), median, and range. Data normality was assessed using the Shapiro-Wilk test. As the data were not normally distributed, the non-parametric Kruskal-Wallis test was used to analyze the accumulation of inorganic elements in all tissues. In order to identify outliers in data sets that may include both biological variability and analytical noise, the Robust Regression and Outlier Removal (ROUT) method was applied. The Mann-Whitney *U* test was used to analyze the influence of age, sex and spatial variation in metal(loid) accumulation, as well as oxidative stress parameters. Spearman’s test was used to assess the different correlations between metal levels and tissues as well as oxidative stress biomarkers. The strength of the correlations, calculated using Spearman’s rank correlation coefficient (*r*), was interpreted following the criteria established by Kalisińska et al. (2023): *r* = 0.8–1 very strong; 0.6–0.79 strong; 0.4–0.59 moderate, 0.2–0.39 weak, 0–0.19 very weak. The significance level was established as *p* < 0.05.

## Results

### Heavy metal concentrations

Heavy metal(loid) concentrations (As, Cd, Hg, Pb, and Zn) in European otters from NW Spain were assessed. Table 1 shows the main descriptive statistics of metal(loid) concentrations (mg kg^−1^ dw) in hair, liver, and kidney. A Kruskal-Wallis test revealed significant differences between all heavy metals in all tissues in the medians among the 15 groups (*H* = 348.1, *df* = 14, *p* < 0.0001). The effect size (*η*^2^ = 0.84) indicates that 84.1% of the variance in the ranks is attributable to group differences. Hg and Zn concentrations were significantly higher in the liver and hair (*H* = 9.805, *df* = 2, *p* = 0.0074, *η*^2^ = 0.12; *H* = 21.62, *df* = 2, *p* < 0.0001, *η*^2^ = 0.26), whereas As and Cd were mainly found in the liver and kidney (*H* = 39.59, *df* = 2, *p* < 0.0001, *η*^2^ = 0.48; *H* = 39.70, *df* = 2, *p* < 0.0001, *η*^2^ = 0.48). Pb concentrations were higher in hair than in the liver or kidney (*H* = 12.38, *df* = 2, *p* = 0.0021, *η*^2^ = 0.15). Next, the ROUT method was applied, and three outliers were identified: two adult and one juvenile females with an excessive amount of Hg in the liver (290.1, 218.1, and 402.9 mg kg^−1^ dw).

**Table 1:** Descriptive statistics corresponding to As, Cd, Hg, Pb and Zn concentrations in European otters from NW of Spain. Data are expressed in mg kg^−1^ dry weight (dw)

	Element	Mean ± SEM	SD	Median	Range
Hair	As	0.244 ± 0.04	0.19	0.18	0.047–0.634
	Cd	0.02 ± 0.003	0.02	0.02	0.004–0.054
	Hg	55.09 ± 6.86	36.3	57.0	1.025–142.8
	Pb	1.307 ± 0.62	3.29	0.47	0.075–17.58
	Zn	133.6 ± 6.20	32.8	145	72.63–186.9
Liver	As	3.644 ± 0.75	3.87	2.59	0.094–17.37
	Cd	1.153 ± 0.28	1.47	0.59	0.005–5.780
	Hg	39.36 ± 5.82	28.53	41.3	0.406–94.47*
	Pb	0.275 ± 0.06	0.29	0.15	0.038–1.184
	Zn	154.8 ± 17.5	90.9	108	55.83–377.5
Kidney	As	3.272 ± 0.62	3.27	2.28	0.110–11.25
	Cd	1.813 ± 0.35	1.83	1.29	0.006–6.950
	Hg	29.22 ± 4.49	23.8	26.8	0.637–113.9
	Pb	0.265 ± 0.04	0.19	0.21	0.048–0.837
	Zn	86.01 ± 6.29	33.3	76.8	53.42–208.6

*SEM* standard error of mean, *SD* standard deviation

*Outliers (290.1, 218.1 and 402.9 mg kg^−1^ dw) were excluded

### Correlation study

**Fig. 2:**
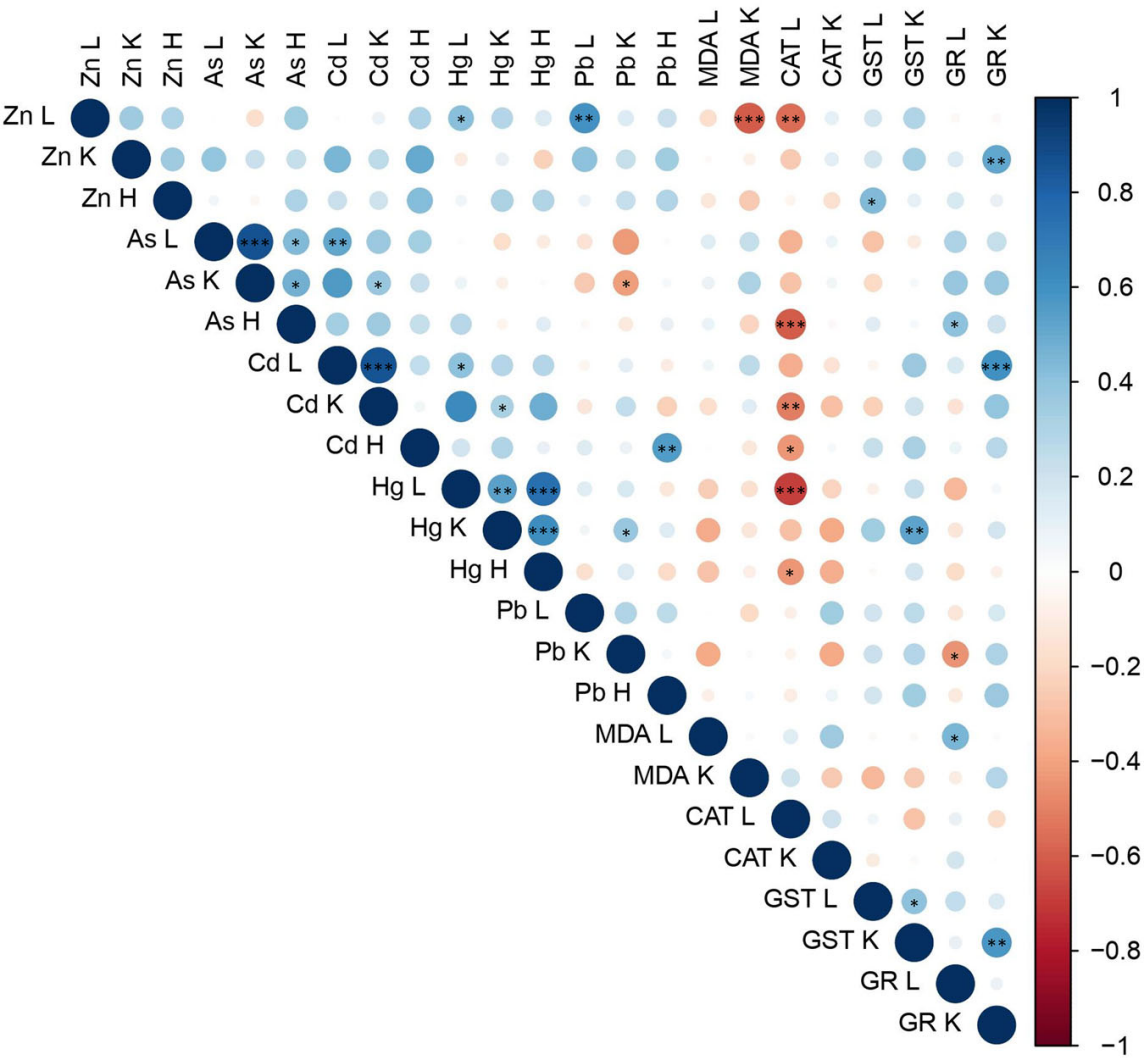
Correlation matrix of the variables (heavy metal concentrations and oxidative stress biomarkers) analyzed in the hair (H), liver (L), and kidney (K) of European otters. Blank squares represent no statistically significant correlation. Blue circles represent a positive correlation. Red circles represent a negative correlation. The size of the circle represents the correlation coefficient. * (*p* < 0.05), ** (*p* < 0.005), *** (*p* < 0.0001)

When assessing the suitability of hair as an alternative tool for comparing internal metal(loid) concentrations, we observed a strong correlation between Hg in the hair and the liver (Spearman test: *r* = 0.75, *p* < 0.0001) and the kidney (*r* = 0.62, *p* < 0.001,) (Fig. 2). There was a moderate correlation in As concentration between hair and liver (*r* = 0.44, *p* = 0.022), and hair and kidney (*r* = 0.48, *p* = 0.011). The association between organs was observed, As and Cd concentrations in the liver were strongly correlated with those in the kidney (*r* = 0.87; *r* = 0.86, *p* < 0.0001). However, the correlation between Hg concentrations in the liver and kidney was moderate (*r* = 0.54, *p* = 0.004). When the interaction between metal(loid)s were analyzed, we observed a moderate correlation between Hg and Zn (*r* = 0.42, *p* = 0.031), Hg and Cd (*r* = 0.41, *p* = 0.034), Pb and Zn (*r* = 0.59, *p* = 0.001), and As and Cd (*r* = 0.51, *p* = 0.006) in the liver. In the kidney, there was a weak correlation between As and Cd (*r* = 0.39, *p* = 0.037), As and Pb (*r* = −0.38, *p* = 0.048), and Cd and Hg (*r* = 0.39, *p* = 0.036), and a moderate correlation between Hg and Pb (*r* = 0.43, *p* = 0.023), while in the hair we only found a moderate association between Cd and Pb (*r* = 0.59, *p* = 0.001).

### Intrinsic and extrinsic factors

**Fig. 3:**
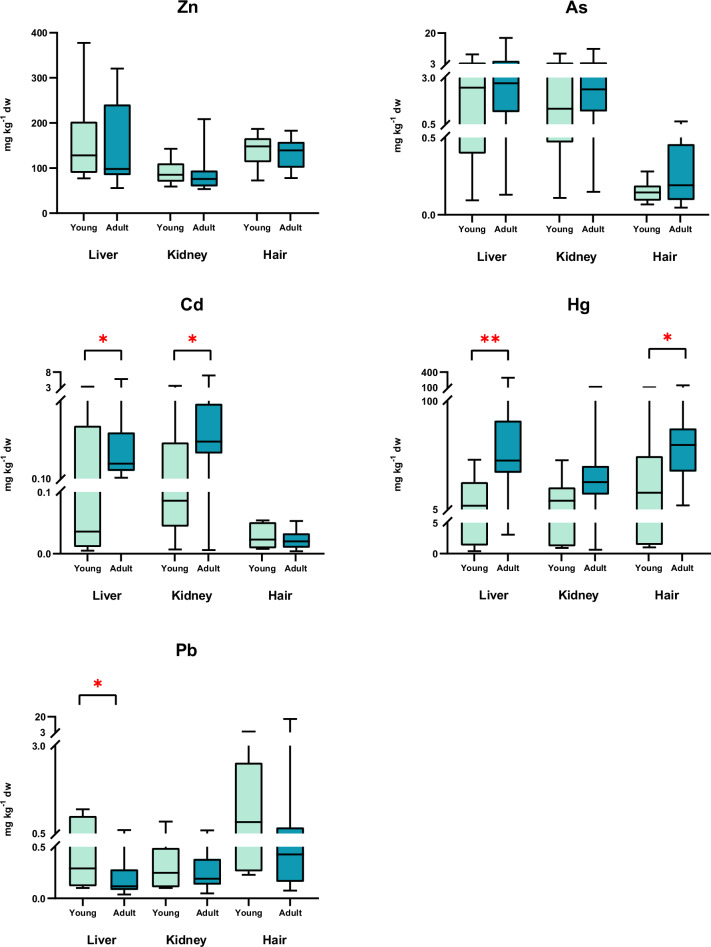
Distribution of Hg, Cd, Pb, and Zn (mg kg^−1^ dw) in the liver, kidney, and hair of European otters, according to age. Box plots represent median values and 25–75 percentiles. Significant levels were * *p* < 0.05, ** *p* < 0.005.

An apparent age-related accumulation of heavy metal(loid)s in each tissue was observed. Cd, Hg and Pb was significantly higher in adults than in juvenile otters (Fig. 3). Outliers were identified and removed from the comparative study to improve data trend models, reduce variance, and minimize the risk of drawing erroneous conclusions. In the comparative study, we showed that liver and hair Hg concentrations were significantly higher in adults (68.21 and 62.72 mg kg^−1^ dw, respectively) compared to juveniles (14.77 and 32.21 mg kg^−1^ dw, respectively) (U = 16, *p* = 0.0025, *η*^2^ = 0.34; U = 32, *p* = 0.0271, *η*^2^ = 0.23, in liver and hair, respectively). Similar results were observed for Cd accumulation in the liver (1.279 and 0.7916 mg kg^−1^ dw for adults and juveniles, respectively) and kidney (2.156 and 0.7823 mg kg^−1^ dw for adults and juveniles, respectively) (U = 34, *p* = 0.0478, *η*^2^ = 0.21; U = 32, *p* = 0.0271, *η*^2^ = 0.25). There were no statistically significant differences in As concentrations between adults and juveniles. Pb concentration in liver samples was significantly higher in juveniles (0.4959 mg kg^−1^ dw) than in adults (0.1978 mg kg^−1^ dw) (U = 34, *p* = 0.0478, *η*^2^ = 0.18). Juveniles also showed high Zn concentrations in all tissues, but there were no statistically significant differences in comparison with adults (Table S1).

**Fig. 4:**
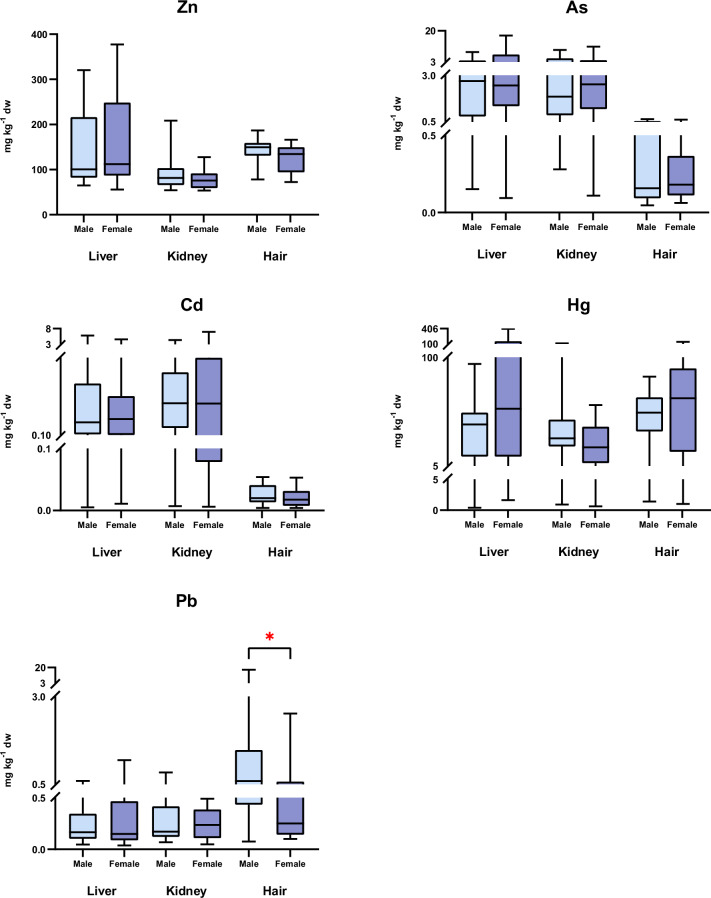
Distribution of Hg, Cd, Pb, and Zn (mg kg^−1^ dw) in the liver, kidney, and hair of European otters, according to sex. Box plots represent median values and 25–75 percentile. Significant levels were * *p* < 0.05

When the comparative study was carried out according to sex, statistically significant differences were observed in the concentration of Pb in the hair, with higher levels in males (2.143 mg kg^−1^ dw) than in females (0.4721 mg kg^−1^ dw) (U = 49, *p* = 0.0241, *η*^2^ = 0.25) (Fig. 4). Females tended to accumulate a higher amount of metal(loid)s in the liver than males, while an opposite trend was observed in the hair (Table S2).

Spatial variation was assessed as an extrinsic factor. However, due to the small number of otter samples from the inland (*n* = 3) compared to those from coastal areas (*n* = 25), our ability to conduct an effective statistical analysis was limited. This limitation made it difficult to draw robust conclusions about the distribution and variability of the data relative to inland or coastal areas. In any case, otters inhabiting coastal areas exhibited higher Cd and As concentrations in all tissues than inland otters, with significantly higher Cd levels observed in hair (U = 6, *p* = 0.0131, *η*^2^ = 0.37). This pattern was observed for almost all inorganic elements but with no statistically significant differences (*p* > 0.05) (Table S3).

### Oxidative stress biomarkers

Descriptive statistics of oxidative stress biomarkers, enzyme activity (CAT, GR, and GST), and lipid peroxidation (MDA in the liver and kidney) are summarized in Table 2. There was a higher level of CAT (Mann-Whitney *U* test: U = 173, *p* = 0.0007, *η*^2^ = 0.48) and GR activity in the liver relative to the kidney, but this difference was not statistically significant in GR (*p* > 0.05). There were no significant differences in GST and MDA activity between the liver and the kidney.

**Table 2:** Main descriptive statistics corresponding to enzyme activities and lipid peroxidation in liver and kidney samples of European otters from NW of Spain

	Biomarker	Mean ± SEM	SD	Median	Range
Liver	CAT	1.872 ± 0.45	2.35	1.10	0.031–11.29
	GR	0.030 ± 0.003	0.01	0.02	0.017–0.065
	GST	6.282 ± 0.83	4.31	6.09	0.352–19.48
	MDA	0.172 ± 0.01	0.06	0.16	0.082–0.280
Kidney	CAT	0.768 ± 0.19	1.01	0.30	0.005–4.056
	GR	0.028 ± 0.002	0.01	0.03	0.009–0.624
	GST	7.504 ± 0.84	4.44	6.21	2.471–22.53
	MDA	0.286 ± 0.07	0.37	0.16	0.081–1.872

CAT, GR, and GST (mU mg^−1^ protein)

MDA (nmol mg^−1^ protein)

The correlation between oxidative stress biomarkers in the liver and kidney was only moderately positive for GST (*r* = 0.41, *p* = 0.0356). We identified a correlation between heavy metals and various oxidative stress biomarkers (Fig. 2). Specifically, in the liver, CAT activity showed a strong negative correlation with Hg concentrations (*r* = −0.69, *p* < 0.0001) and a moderate negative correlation with Zn (*r* = −0.55, *p* = 0.002). In contrast, in kidney samples, there was a moderate positive correlation between GR activity and Zn concentrations (*r* = 0.52, *p* = 0.005), and between GST activity and Hg concentrations (*r* = 0.53, *p* = 0.0008). We observed a moderate positive correlation between MDA levels and GR activity in the liver (*r* = 0.46, *p* = 0.016), and between GST and GR activities in the kidney (*r* = 0.50, *p* = 0.0064). Age, sex and habitat did not influence the oxidative stress biomarkers (*p* > 0.05) (Tables S4 and S5).

## Discussion

### Heavy metal burden in hair, liver and kidney

This biomonitoring study quantified metal(loid) concentrations (As, Cd, Hg, Pb, Zn) in European otters from northwestern Spain, providing the first report of metal(loid) accumulation using otter hair as a non-invasive biomonitoring matrix. A high mean concentration of Hg was recorded in the hair of otters. This concentration (55.09 mg kg^−1^ dw) is well above the normal background levels established in the hair of North American river otters (*L. canadiensis*, 1–5 mg kg^−1^) (Sheffy and St. Amant, 1982). Previous studies have reported the use of feces as non-invasive tools for assessing heavy metals in otters from the Iberian Peninsula (Rodríguez-Estival et al. 2020; Baos et al. 2022). Hair is considered a suitable matrix for Hg determination due to the metal’s strong affinity for keratin, and also because it reflects long-term exposure that may lead to chronic effects (Rashed and Soltan, 2005; García-Muñoz et al. 2023b). Compared to previous studies conducted on European otters from central and northern Europe, our results showed significantly higher total Hg levels in the hair of Iberian otters (Table 3). In mammals, neurotoxic effects have been reported at hair Hg levels of 6 and 10 mg kg^−1^ (Ferrante et al. 2018). More than 80% of the otters in this study exceeded these critical threshold values. Moreover, 75% of our specimens exceeded both the lowest observed adverse effect level (LOAEL) for Hg in the hair of mammalian wildlife, and the threshold value in hair associated with neuronal effects in mustelids established at 30 mg kg^−1^ (Kalisińska et al. 2019). Some literature suggests that the average hair Hg concentration in piscivorous mammals is between 5 and 15 ppm. Our findings surpassed this range by four times (Kalisińska et al. 2019). The highest recorded level of mercury in the hair of semi-aquatic mustelids was reported in American otter, reaching 234 mg kg^−1^ (Yates et al. 2005). Our data showed a maximum value of 142.8 mg kg^−1^.

**Table 3:** Literature of Europe studies concerning As, Cd, Hg, Pb and Zn concentration in hair, liver, and kidney of European otter (*Lutra lutra*). Mean, Range

Site *(n)*		As	Cd	Hg	Pb	Zn	*Ref*.
*Hair*							
NW Spain	dw	0.244 (0.047–0.634)	0.02 (0.004–0.054)	55.09 (1.025–142.8)	1.307 (0.075–17.58)	133.6 (72.63–186.9)	Present study
Slovakia (22)	dw			10.6–12.9 (0.4–20.1)			Pitoňáková et al. (2023)
Denmark (117)	dw			12.62 (0.297–40.0)			Dibbern et al. (2021)
Finland (47)	dw			19			Lodenius et al. (2014)
Finland (39)	dw		0.005–0.038	5.9–29.5		76.4–95.4	Hyvärinen et al. (2003)
UK^a^	dw		0.77	2.38	1.28		Madsen and Mason (1987)
UK (25)	dw			18.75 (1.3–85.1)			Mason et al. (1986)
*Liver*							
NW Spain	dw	3.644 (0.094–17.37)	1.153 (0.005–5.78)	39.36 (0.406–94.47)	0.275 (0.038–1.184)	154.8 (55.83–377.5)	Present study
Slovakia (23)	dw			4.3 (0.4–16.8)			Pitoňáková et al. (2023)
Denmark (117)	ww			2.57 (0.015–10.1)			Dibbern et al. (2021)
NW Poland (8)	dw			7.815 (2.358–12.473)			Kalisińska et al. (2021)
England and Wales (278)	dw	0.275 (0.003–9.38)	0.444 (0.002–17.1)	6.82 (0.047–50.3)	0.571 (0.06–9.57)	118 (50.0–465)	Brand et al. (2020)
Southern Italy (10)	ww	0.021–0.196	0.045–0.152		0.038–0.174	10.74–53.12	Esposito et al. (2020)
France (65)	dw		0.26 (0.05–1.77)	4.68 (0.17–18.52)	1.02 (0.05–9.30)		Alomar et al. (2016)
Finland (96)	ww		0.08 (0.01–0.3)(n = 39)	5.7	0.1–0.2 (n = 19)	36 (19–75)(n = 19)	Lodenius et al. (2014)
England and Wales (50)	dw	0.094 (0.067–0.182)	0.167 (0.068–0.683)	4.955 (2.840–9.640)		102 (85.20–125.0)	Walker et al. (2011)
France (20)	dw	0.1 (nd-0.4)	0.3 (0.1–1.0)	2.1 (0.4–8.1)	1.0 (0.3–7.3)		Lemarchand et al. (2010)
Hungary (111)	dw		0.15 (nd-1.168)	3.85 (nd-29.54)	0.213 (nd-1.383)	99.75 (41.49–368.13)	Lanszki et al. (2009)
Finland (31)	ww		0.10–0.60	1.2–5.5		101.0–135.9	Hyvärinen et al. (2003)
Austria, Czech Republic, and Hungary (27)	dw		0.31–1.51 (0.01–5.42)		0.37–0.83 (0.01–2.2)		Gutleb et al. (1998)
Denmark (69)	ww			2.30 (0.03–12.37)			Mason and Madsen (1992)
UK (19)	ww			5.37 (1.2–20.5)			Mason et al. (1986)
Southern Spain (5)	ww		0.19 (0.13–0.28)	8.28 (3.92–17.48)	0.73 (0.69–0.78)	40.09 (31.6–50.9)	Hernandez et al. (1985)
*Kidney*							
NW Spain	dw	3.272 (0.11–11.25)	1.813 (0.006–6.95)	29.22 (0.637–113.9)	0.265 (0.048–0.837)	86.01 (53.42–208.6)	Present study
Slovakia (22)	dw			3.3 (0.6–9.8)			Pitoňáková et al. (2023)
NW Poland (8)	dw			5.304 (2.612–7.584)			Kalisińska et al. (2021)
Southern Italy (10)	ww	0.021–0.22	0.058–1.23		0.031–0.676	12.95–35.62	Esposito et al. (2020)
Finland (38)	ww			1.6			Lodenius et al. (2014)
Finland (31)	ww		0.39–1.84	0.4–1.4			Hyvärinen et al. (2003)
Austria, Czech Republic, and Hungary (27)	dw		0.32–0.91 (0.03–4.6)		0.23–1.12 (0.02–3.7)		Gutleb et al. (1998)
UK (16)	ww			2.27 (1.35–6.79)			Mason et al. (1986)

All data are expressed in mg kg^−1^, μg g^−1^ (part per million, ppm)

*n* number of samples analysed, *nd* non-detected

Range was provided when data was available

^a^Data are not available

Metal bioaccumulation primarily occurs in detoxification organs, liver and kidney (Lentini et al. 2017; Mayack, 2012). In line with the hair results, the levels of Hg in both organs were surprisingly high. The concentrations of Hg in the liver exceeded the reference lethal concentration ( > 25 mg kg^−1^ ww) in 21% of otters. Additionally, the LOAEL related to clinical signs and death, reported for the liver and kidney of mustelids (18.1 mg kg^−1^ ww), was also exceeded in 25% of our samples (Wobeser et al. 1976). Here we report that three individuals exhibited such high Hg concentrations in liver considered as outliers. Two outliers came from Marín (Pontevedra) (with Hg concentrations of 218.1 and 402.9 mg kg^−1^ dw). Previous research has shown that the Rias of Pontevedra is suffering a significant pollution issue, characterized by the persistent presence of Hg (Beiras et al. 2002; Vizuete et al. 2023). This may be attributed to the presence of a chlorine-alkali plant in this area, where Hg is the main pollutant released into the environment.

Our results are consistent with data from a study that assessed Hg levels in mussels from the same area, one of the otter’s main food sources (Besada et al. 2011). These authors attributed the Hg levels to the same industry and a paper pulp factory. The third outlier (290.1 mg kg^−1^ dw) came from northern Galicia, where prominent aluminium and alumina factories, as well as a large thermal power plant, account for 49% of the total emissions of Hg in the region (Nóvoa-Muñoz et al. 2008; Giráldez et al. 2022). Mason and Macdonald (1988) pointed out that heavy metal pollution affecting the food supply was responsible for the decline of the European otter in England. Moreover, 82% of our liver samples exceeded the level that indicates possible environmental contamination by Hg (1.1 mg kg^−1^ ww), as well as the background Hg concentration values for piscivorous mustelids from Europe (<2.0 in the liver and < 1.5 mg kg^−1^ ww in the kidney) (Eisler, 1987; Kalisińska et al. 2021). These concentrations were higher than those in similar studies from across Europe (ranging 0.03 to 50.3 mg kg^−1^ and 0.4 to 7.58 mg kg^−1^ dw, in liver and kidney, respectively) (Table 3). The liver Hg concentrations that we report here were higher than those reported for otters from North America ( < 4–5 mg kg^−1^ ww) (Wren, 1986; Kalisińska et al. 2021) and from a Protected National Park in the south of Spain (8.28 mg kg^−1^ ww) (Hernández et al. 1985).

Significant levels of Pb were detected in otter hair, probably due to its known affinity for keratin (Kales and Christiani, 2005). These results are in agreement with the few existing studies, including those of Madsen and Mason (1987). Cd concentrations in hair were similar to those recorded in Finland (Hyvärinen et al. 2003), but lower than those in the UK (Madsen and Mason, 1987). Although data on As concentrations in otter hair are lacking, the low levels detected in this study are consistent with those recorded in other Mediterranean mustelids, such as the Eurasian badger (*Meles meles*) and marten (*Martes foina*) (Squadrone et al. 2022; Trossi et al. 2023). The molting process represents a valuable opportunity for non-invasive sampling and metal determination without causing harm to the animal. Toxicokinetic studies have shown that the bloodstream supplies metal(loid)s to the hair, which are continuously excreted in considerable amounts and stored in the hair follicle as it grows (Hyvärinen et al. 2003; Vermeulen et al. 2009).

Kidney is the primary target organ affected by Cd and Pb accumulation, with the liver serving as a secondary site of accumulation when the kidney’s capacity is exceeded (Cooke, 2011; Ma, 2011). We detected Cd and Pb in all tissue samples, albeit at significantly low levels. The mean Cd and Pb concentrations were significantly below the levels known to cause detrimental effects in mammals (105 mg kg^−1^ dw and 100 mg kg^−1^ ww in kidney and liver, and 15 and 5 mg kg^−1^ dw in kidney and liver, respectively) (Shore and Douben, 1994; Gutleb et al. 1998; Cooke, 2011; Ma, 1996, 2011; Baranowska-Bosiacka et al. 2019). Compared to other biomonitoring studies, the observed Cd and Pb concentrations were in the range quantified in the liver and kidney of otters from central and northern Europe (Table 3).

For As, we found that the liver and kidney accumulated similar levels. Although the kidney is the primary organ for As excretion, and the liver is the main organ in storing it (Pereira et al. 2006). This was evidenced by the maximum As values obtained in the present study (Binkowski, 2019). About 36% of our liver samples were above the threshold of 3 mg kg^−1^, which is the As background limit with no adverse effects (Pereira et al. 2006). Additionally, liver As concentration was within the range reported in other studies of European otters (0.003–9.38 mg kg^−1^). These results were markedly higher than those obtained in other mustelids from the same region (e.g., the Eurasian badger) (García-Muñoz et al. 2023a). Other authors have assessed the concentrations of As in sea otters (*Enhydra lutris*), reporting liver As concentrations of up to 5.7 μg g^−1^ dw with no apparent toxicological consequences (Kubota et al. 2001).

### Correlation study: non-invasive vs invasive samples

We observed a positive correlation between Hg levels accumulated in the hair and the liver and kidney. Other studies have reported similar correlations in the otters from Europe and North America (Evans et al. 1998; Yates et al. 2005; Klenavic et al. 2008; Strom, 2008; Lodenius et al. 2014; Dibbern et al. 2021). Sheffy and St. Amant (1982) reported a ratio of approximately 2.5:1 for Hg levels in hair, relative to the kidney and the liver. In our study, the observed ratios were 1.3:1 for the liver and 1.9:1 for the kidney.

Correlations between As levels and hair and internal organs had never been previously reported in otters. These positive correlations highlight the potential use of hair as a non-invasive tissue to accurately represent metal accumulation in wild organisms. Other studies reported a positive correlation in Hg, As, and Cd levels between the liver and kidney, which is possibly explained by similar detoxification dynamics, and high reabsorption and blood transfer rates (Boening, 2000; Lentini et al. 2017). Zn plays a significant role in the structure of metalloenzymes, and elements such as Pb or Hg may influence Zn metabolism. The correlation between Zn, Hg, and Pb in both organs was expected based on previous studies (Mason and MacDonald, 1986; Lanszki et al. 2009). It is suggested that the toxicity and bioaccumulation of heavy metal(loid)s rely on the interaction between essential and non-essential metals through complex antagonistic interactions in which elements compete for metallothionein binding sites (López Alonso et al. 2004; Henkel and Krebs, 2004).

### Influence of endogenous and exogenous factors on heavy metal accumulation

Adult otters exhibited a higher accumulation of Hg in hair. It is well known that age can have a significant influence on heavy metal(loid) accumulation in wild mammals (Shore and Rattner, 2001). Because of the low removal rate of Hg, it was expected that its concentration in hair would increase with age due to prolonged exposure to a contaminated environment (Ben-David et al. 2001). Other studies have reported a similar correlation between hair Hg concentration and otter age (Hyvärinen et al. 2003; Yates et al. 2005; Dibbern et al. 2021). In the liver and kidney, there was a higher accumulation of Hg and Cd in adults relative to juveniles, which is in keeping with previous studies on European otters (Mason and Madsen, 1992; Lanszki et al. 2009; Lemarchand et al. 2010; Walker et al. 2011; Lodenius et al. 2014; Alomar et al. 2016). Brand et al. (2020) attributed this difference to dietary habits, with older otters predominantly consuming large fish, while young otters feed mainly on crustaceans.

Pb had a higher accumulation in the liver of young otters than in adults, possibly because of increased absorption and retention of calcium (Skerfving and Bergdahl, 2015). We also found a higher accumulation of Zn in liver, kidney and hair of juveniles. Previous studies have reported the vulnerability of juveniles to Zn accumulation because they absorb it more efficiently (Kosik-Bogacka and Łanocha-Arendarczyk, 2019). Sexual dimorphism influences behavioral habits and diet, and could explain the accumulation of Pb in males. For instance, larger males capture larger prey, which would represent a higher Pb intake (Chételat et al. 2020). Previous studies have shown that males accumulate higher concentrations of heavy metals compared to females (Hyvärinen et al. 2003; Lemarchand et al. 2010; Esposito et al. 2020). Lower Hg levels in females have been attributed not only to elimination through placental and lactational routes, but also to differences in movement patterns, as females usually tend to have smaller home ranges than males and are less exposed to a reduced range of contaminants (Chételat et al. 2020; Sanders et al. 2020). Nevertheless, opposite results have also been reported, with female otters tending to accumulate higher amounts of Hg compared to males (Klenavic et al. 2008; Mayack, 2012; Lodenius et al. 2014; Dibbern et al. 2021). Finally, Hyvärinen et al. (2003) reported that adult male otters have a higher molting rate than females, which could explain the lower metal levels observed in males. The small sample size and its unequal composition between sexes and age groups could have limited the ability of the study to detect certain differences, especially in comparative analyses. However, despite these limitations, the effect sizes were consistent regarding the accumulation of metal(loid)s in the different tissues, and the influence of endogenous factors. This indicates that, although statistical power may have been low due to the small sample size, the observed differences are biologically relevant and reflect consistent patterns in the population studied.

In the present study, we could not compare otters from different habitats, due to the small number of inland otters. However, we found higher accumulation of metal(loid)s in otters from coastal areas compared to inland regions. This may be explained by the heavy metal background levels reported in the western region of Galicia (Giráldez et al. 2022). Therefore, otters residing in coastal areas may have been exposed to greater environmental pressures than inland otters, owing to the enhanced presence of heavy metal(loid)s. In contrast, Dibbern et al. (2021) reported that hepatic Hg concentration increased with distance from the marine coastline. Klenavic et al. (2008) and Mayack (2012) also reported higher metal concentrations in otters inhabiting rivers compared to those from coastal regions.

### Oxidative stress biomarkers

The effect of size on oxidative stress biomarkers was moderate to strong. Here we reported correlations between metal(loid)s and antioxidants, specifically CAT was associated with Hg and Zn, GR with Zn, and GST with Hg. CAT is an antioxidant enzyme which plays a role in converting hydrogen peroxide into water and oxygen, particularly when hydrogen peroxide levels are high (Halliwell and Gutteridge, 2007). The decrease in CAT activity could be attributed to inhibition induced by metal toxicity, especially high levels of Hg. Low CAT activity has also been exhibited in other wildlife mammals such as bats exposed to metal(loid)s in mining areas (Ruiz et al. 2019; Gil-Jiménez et al. 2021). The positive relationship found between GR and Zn could be due to this antioxidant’s responsibility for maintaining the homeostasis of GSH, a tripeptide that plays a central role in detoxification processes (Isaksson, 2010). Given that Zn is an essential metal involved in the control of oxidative stress and acts as a cofactor for multiple enzymes, including GR, it is reasonable to expect an association between both GR and Zn (Marreiro et al. 2017). GST is a phase II enzyme responsible for cellular detoxification of xenobiotics and has a crucial function in protecting against toxic chemicals (Viegas-Crespo et al. 2003). The modulation of GST activity is dependent on the degree of contamination and chemical forms. A positive correlation between GST activity and Hg in the kidney was observed. An increase in the levels of this enzyme may result from the elimination of oxidant compounds generated by metal pollutants, which could explain its correlation with Hg concentrations (Viegas-Crespo et al. 2003). It is worth noting that the individual with the highest Hg concentration in the kidney exhibited one of the highest GST activities. Here, we provide evidence that high Hg levels induce oxidative stress responses, manifested by the inhibition of CAT and an increase in GST activity. MDA may be a valuable biomarker of exposure to pollutants as it is a final product of lipid peroxidation and an indicator of oxidative stress (Funes et al. 2006); however, we did not find any correlation between metal concentrations and MDA. MDA was positively correlated with GR activity in the liver; thus, higher lipid peroxidation levels may induce the activation of antioxidants to scavenge ROS (Halliwell and Gutteridge, 2007). When analyzing endogenous and exogenous factors on oxidative stress biomarkers, we did not observe any statistically significant influence. This study is the first contribution to establishing a baseline of the oxidative status in otters, which will serve as a reference point for future research.

It must be acknowledged that this study presented several limitations, mainly related to the sample size, which was limited by the limited availability of recently deceased otters from wildlife recovery centers. To reduce the impact of post-mortem degradation on oxidative stress biomarkers, only individuals that had recently died were included. However, reliance on opportunistic samples, such as roadkill, may introduce variability in the results. Although significant correlations were found between heavy metal concentrations and oxidative stress biomarkers, these associations do not prove causality. Confirmation of causal links would require controlled exposure experiments. Furthermore, because the samples represent a subset of the population, physiological conditions related to the cause and timing of death may affect biomarker levels independently of contaminant exposure.

In summary, our results suggest that otter hair may reflect environmental exposure to heavy metals, supporting its potential as a non-invasive biomonitoring tool. Given the near-threatened status of *Lutra lutra*, the use of hair could offer a minimally invasive and sustainable alternative for future monitoring efforts. Although the Water Framework Directive (Directive 2000/60/EC) sets strict limits for surface water pollutants, elevated mercury concentrations were detected in otters from industrial areas. This is consistent with recent reports indicating that 24% of rivers and coastal waters in Galicia show signs of poor ecological status (Gaia, 2024). Although the observed correlations between oxidative stress biomarkers and metal(loid) concentrations may reflect physiological responses to environmental pollution, further studies are needed to confirm these associations and clarify possible causal mechanisms. Overall, these findings provide a preliminary baseline and reinforce the importance of coordinated efforts to monitor and mitigate pollution, consistent with the “One Health” approach.

## Supplementary information

Supplementary Material

## Data Availability

No datasets were generated or analysed during the current study.
