# Supplementary material for: First study of heavy metals analysis in hair and oxidative status of European Otters (Lutra lutra) from Southwestern Europe
Source: Ecotoxicology. 2025 Jul 1;34(8):1351–67. doi: 10.1007/s10646-025-02911-x (PMC12476393; doi:10.1007/s10646-025-02911-x)
Supplement: Supplementary file 1 — Supplementary Material [file 10646_2025_2911_MOESM1_ESM.docx]

**Supplementary material**

**First study of heavy metals analysis in hair and oxidative status of European otter (*Lutra lutra*) from SW of Europe**

Javier García-Muñoz, David Fernández Casado, Ángel Portillo Moreno, María del Prado Míguez-Santiyán^a^, Francisco Soler Rodríguez^a^, Ana López-Beceiro^b^, Luis Eusebio Fidalgo^b^, Salomé Martínez-Morcillo^a^, Marcos Pérez-López^a^

^a^ Toxicology Area, Faculty of Veterinary Medicine (UEX), 10003 Caceres, Spain

^b^ Department of Veterinary Clinical Sciences, Faculty of Veterinary Medicine (USC), 27003 Lugo, Spain

Corresponding author: Javier García Muñoz (*jgarciamz@unex.es*)

Postal address: Toxicology Area, Faculty of Veterinary Medicine (UEX), 10003 Cáceres, Spain.

**Table S1.** Main descriptive statistics corresponding to As, Cd, Hg, Pb and Zn concentrations in otters according to the age influence. Data are expressed in milligrams per kilograms (mg kg^-1^) dry weight (dw).

|  |  |  | As | Cd | Hg | Pb | Zn |
| --- | --- | --- | --- | --- | --- | --- | --- |
| Young  (*n* = 7) | Hair | Mean ± SEM | 0.15±0.03 | 0.03±0.01 | 32.2±14.3**^A^** | 1.34±0.50 | 140.3±14.1 |
|  |  | Min | 0.07 | 0.01 | 1.03 | 0.30 | 72.6 |
|  |  | Max | 0.28 | 0.05 | 104.8 | 3.75 | 186.9 |
|  | Liver | Mean ± SEM | 2.87±1.05 | 0.79±0.51**^A^** | 14.8±7.61**^A^** | 0.50±0.16**^A^** | 166.2±39.1 |
|  |  | Min | 0.09 | 0.01 | 0.41 | 0.10 | 77.0 |
|  |  | Max | 8.27 | 3.32 | 48.5 | 1.18 | 377.5 |
|  | Kidney | Mean ± SEM | 2.59±1.12 | 0.78±0.51**^A^** | 15.9±6.60 | 0.34±0.10 | 90.5±10.6 |
|  |  | Min | 0.11 | 0.01 | 0.92 | 0.10 | 59.3 |
|  |  | Max | 8.69 | 3.59 | 48.1 | 0.84 | 142.9 |
| Adult  (*n* = 21) | Hair | Mean ± SEM | 0.2747±0.05 | 0.02±0.003 | 62.7± 7.29**^B^** | 1.30±0.82 | 131.4±6.95 |
|  |  | Min | 0.05 | 0.004 | 8.27 | 0.08 | 77.8 |
|  |  | Max | 0.63 | 0.05 | 142.8 | 17.58 | 182.7 |
|  | Liver | Mean ± SEM | 3.91±0.94 | 1.28±0.34**^B^** | 68.2±15.5**^B^** | 0.20± 0.04**^B^** | 150.8±19.8 |
|  |  | Min | 0.13 | 0.13 | 3.09 | 0.08 | 55.8 |
|  |  | Max | 17.4 | 5.78 | 290.1 | 0.60 | 320.4 |
|  | Kidney | Mean ± SEM | 3.50±0.74 | 2.16± 0.41**^B^** | 33.7±5.29 | 0.24±0.03 | 84.5±7.70 |
|  |  | Min | 0.15 | 0.01 | 0.64 | 0.05 | 53.4 |
|  |  | Max | 11.3 | 6.95 | 113.3 | 0.58 | 208.6 |

A, B: when letter does not agree, statistically significant differences were observed (p<0.05).

**Table S2.** Main descriptive statistics corresponding to As, Cd, Hg, Pb and Zn concentrations in otters according to the sex influence. Data are expressed in milligrams per kilograms (mg kg^-1^) dry weight (dw).

|  |  |  | As | Cd | Hg | Pb | Zn |
| --- | --- | --- | --- | --- | --- | --- | --- |
| Male  (*n* = 14) | Hair | Mean ± SEM | 0.26±0.06 | 0.03±0.004 | 47.6±6.16 | 2.14±1.21**^A^** | 143.2±8.59 |
|  |  | Min | 0.05 | 0.004 | 1.42 | 0.08 | 78.3 |
|  |  | Max | 0.63 | 0.05 | 83.3 | 17.6 | 186.9 |
|  | Liver | Mean ± SEM | 2.81±0.63 | 1.29±0.44 | 37.7±6.66 | 0.23±0.05 | 146.8±23.2 |
|  |  | Min | 0.15 | 0.005 | 0.41 | 0.05 | 64.7 |
|  |  | Max | 8.27 | 5.78 | 94.5 | 0.59 | 320.4 |
|  | Kidney | Mean ± SEM | 3.15±0.86 | 1.60±0.35 | 35.5±7.34 | 0.28±0.06 | 92.3±11.2 |
|  |  | Min | 0.28 | 0.01 | 0.92 | 0.07 | 54.1 |
|  |  | Max | 9.49 | 4.28 | 113.9 | 0.77 | 208.6 |
| Female  (*n* = 14) | Hair | Mean ± SEM | 0.23±0.05 | 0.02±0.004 | 62.6±12.2 | 0.47±0.17**^B^** | 124.0±8.46 |
|  |  | Min | 0.06 | 0.004 | 1.03 | 0.10 | 72.63 |
|  |  | Max | 0.61 | 0.05 | 142.8 | 2.52 | 166.1 |
|  | Liver | Mean ± SEM | 4.55±1.38 | 1.01±0.36 | 102.2±34.6 | 0.32±0.10 | 163.4±27.1 |
|  |  | Min | 0.09 | 0.01 | 1.63 | 0.04 | 55.8 |
|  |  | Max | 17.4 | 4.56 | 402.9 | 1.18 | 377.5 |
|  | Kidney | Mean ± SEM | 3.40±0.92 | 2.02±0.61 | 22.9±4.87 | 0.25±0.04 | 79.7±5.81 |
|  |  | Min | 0.11 | 0.01 | 1.42 | 0.05 | 53.4 |
|  |  | Max | 11.3 | 6.95 | 58.3 | 0.49 | 127.6 |

A, B: when letter does not agree, statistically significant differences were observed (p<0.05).

**Table S3.** Main descriptive statistics corresponding to As, Cd, Hg, Pb and Zn concentrations in otters from coastal and inland areas. Data are expressed in milligrams per kilograms (mg kg^-1^) dry weight (dw).

|  |  |  | As | Cd | Hg | Pb | Zn |
| --- | --- | --- | --- | --- | --- | --- | --- |
| Coastal  (*n* = 25) | Hair | Mean ± SEM | 0.26±0.04 | 0.03±0.003 **^A^** | 50.6±6.79 | 1.43±0.69 | 131±6.78 |
|  |  | Min | 0.06 | 0.004 | 1.03 | 0.10 | 72.6 |
|  |  | Max | 0.63 | 0.05 | 143 | 17.6 | 187 |
|  | Liver | Mean ± SEM | 3.91±0.78 | 1.20±0.30 | 39.3±6.36 | 0.285±0.06 | 160±18.5 |
|  |  | Min | 0.09 | 0.01 | 0.41 | 0.04 | 55.8 |
|  |  | Max | 17.4 | 5.78 | 94.5 | 1.18 | 378 |
|  | Kidney | Mean ± SEM | 3.59±0.67 | 1.87±0.38 | 30.0±4.89 | 0.26±0.04 | 88.6±6.84 |
|  |  | Min | 0.11 | 0.01 | 0.92 | 0.05 | 53.4 |
|  |  | Max | 11.3 | 6.95 | 114 | 0.84 | 209 |
| Inland  (*n* = 3) | Hair | Mean ± SEM | 0.12±0.04 | 0.007±0.002 **^B^** | 92.3±23.3 | 0.24±0.14 | 154±2.65 |
|  |  | Min | 0.05 | 0.004 | 57.2 | 0.08 | 151 |
|  |  | Max | 0.19 | 0.009 | 136 | 0.52 | 159 |
|  | Liver | Mean ± SEM | 0.34±0.19 | 0.51±0.08 | 40.5±3.75 | 0.15±0.06 | 91.1±0.10 |
|  |  | Min | 0.15 | 0.43 | 36.8 | 0.09 | 91.0 |
|  |  | Max | 0.53 | 0.59 | 44.3 | 0.21 | 91.2 |
|  | Kidney | Mean ± SEM | 0.66±0.27 | 1.37±0.87 | 22.6±11.0 | 0.27±0.10 | 64.2±5.24 |
|  |  | Min | 0.28 | 0.006 | 0.64 | 0.09 | 57.4 |
|  |  | Max | 1.18 | 3.00 | 33.8 | 0.44 | 74.5 |

A, B: when letter does not agree, statistically significant differences were observed (p<0.05).

**Table S4.** Main descriptive statistics corresponding to enzyme activities (nmol min^-1^ mg^-1^ protein) and lipid peroxidation (nmol mg^-1^ protein) in liver and kidney samples of European otters according to age influence.

|  |  |  | MDA | CAT | GST | GR |
| --- | --- | --- | --- | --- | --- | --- |
| Young  (*n* = 7) | Liver | Mean ± SEM | 0.18±0.01 | 1.94±0.42 | 7.09±1.72 | 0.028±0.005 |
|  |  | Min | 0.09 | 0.03 | 1.47 | 0.019 |
|  |  | Max | 0.28 | 3.23 | 14.6 | 0.062 |
|  | Kidney | Mean ± SEM | 0.21±0.03 | 0.97±0.36 | 6.07±1.42 | 0.028±0.005 |
|  |  | Min | 0.11 | 0.01 | 2.58 | 0.012 |
|  |  | Max | 0.35 | 2.42 | 13.9 | 0.055 |
| Adult  (*n* = 21) | Liver | Mean ± SEM | 0.17±0.01 | 1.85±0.60 | 6.00±0.96 | 0.029±0.003 |
|  |  | Min | 0.08 | 0.11 | 0.35 | 0.016 |
|  |  | Max | 0.28 | 11.3 | 19.5 | 0.065 |
|  | Kidney | Mean ± SEM | 0.31±0.09 | 0.70±0.23 | 7.98±1.01 | 0.028±0.002 |
|  |  | Min | 0.08 | 0.005 | 2.47 | 0.008 |
|  |  | Max | 1.87 | 4.06 | 22.5 | 0.062 |

**Table S5.** Main descriptive statistics corresponding to enzyme activities (nmol min^-1^ mg^-1^ protein) and lipid peroxidation (nmol mg^-1^ protein) in liver and kidney samples of European otters according to sex influence.

|  |  |  | MDA | CAT | GST | GR |
| --- | --- | --- | --- | --- | --- | --- |
| Male  (*n* = 14) | Liver | Mean ± SEM | 0.18±0.02 | 2.55±0.81 | 7.38±1.33 | 0.032±0.004 |
|  |  | Min | 0.08 | 0.41 | 2.41 | 0.019 |
|  |  | Max | 0.28 | 11.3 | 19.5 | 0.065 |
|  | Kidney | Mean ± SEM | 0.25±0.07 | 0.65±0.21 | 7.76±0.92 | 0.029±0.004 |
|  |  | Min | 0.11 | 0.01 | 3.23 | 0.012 |
|  |  | Max | 0.97 | 2.42 | 15.1 | 0.062 |
| Female  (*n* = 14) | Liver | Mean ± SEM | 0.16±0.01 | 1.15±0.25 | 5.10±0.90 | 0.026±0.002 |
|  |  | Min | 0.09 | 0.03 | 0.35 | 0.016 |
|  |  | Max | 0.24 | 3.23 | 10.8 | 0.045 |
|  | Kidney | Mean ± SEM | 0.32±0.12 | 0.88±0.32 | 7.25±1.44 | 0.027±0.003 |
|  |  | Min | 0.08 | 0.01 | 2.47 | 0.009 |
|  |  | Max | 1.87 | 4.06 | 22.5 | 0.055 |

**Table S6.** Main descriptive statistics corresponding to enzyme activities (nmol min^-1^ mg^-1^ protein) and lipid peroxidation (nmol mg^-1^ protein) in liver and kidney samples of European otters from coastal and inland areas.

|  |  |  | MDA | CAT | GST | GR |
| --- | --- | --- | --- | --- | --- | --- |
| Coastal  (*n* = 25) | Liver | Mean ± SEM | 0.18±0.01 | 1.49±0.29 | 5.83±0.71 | 0.030±0.003 |
|  |  | Min | 0.09 | 0.03 | 0.35 | 0.016 |
|  |  | Max | 0.28 | 6.91 | 14.6 | 0.065 |
|  | Kidney | Mean ± SEM | 0.30±0.08 ^A^ | 0.69±0.16 | 7.49±0.94 | 0.029±0.002 |
|  |  | Min | 0.08 | 0.01 | 2.47 | 0.011 |
|  |  | Max | 1.87 | 2.42 | 22.5 | 0.062 |
| Inland  (*n* = 3) | Liver | Mean ± SEM | 0.07±0.04 | 6.62±4.67 | 12.0±7.52 | 0.023±0.0002 |
|  |  | Min | 0.08 | 1.95 | 4.43 | 0.022 |
|  |  | Max | 0.14 | 11.3 | 19.5 | 0.023 |
|  | Kidney | Mean ± SEM | 0.15±0.03 ^B^ | 1.46±1.30 | 7.63±0.34 | 0.017±0.005 |
|  |  | Min | 0.11 | 0.01 | 6.96 | 0.008 |
|  |  | Max | 0.21 | 4.06 | 8.04 | 0.028 |

A, B: when letter does not agree, statistically significant differences were observed (p<0.05).


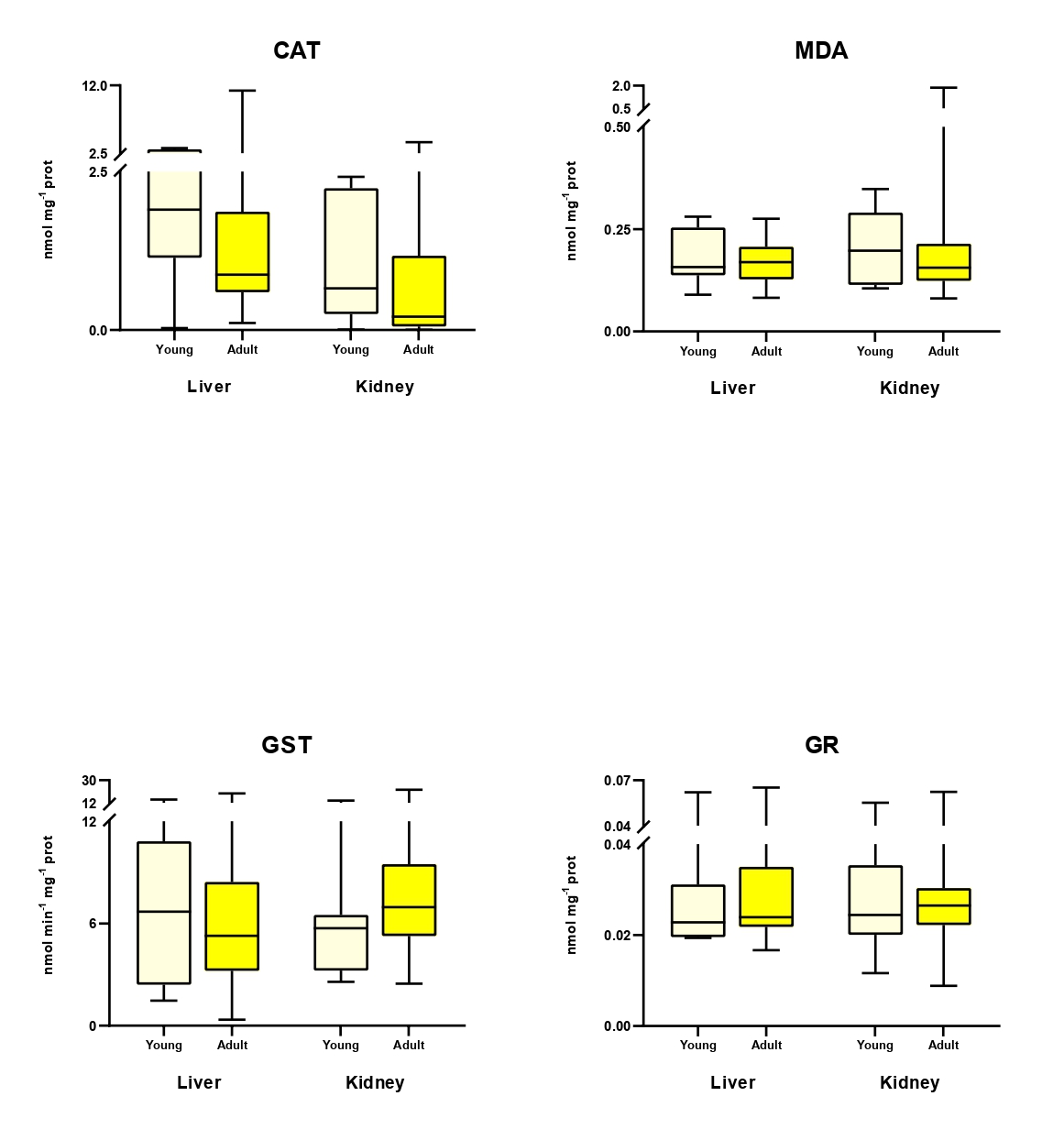


**Fig. S1**. CAT, MDA, GST and GR levels in liver and kidney of European otter according to age. Box plots represent median values and 25-75 percentiles.


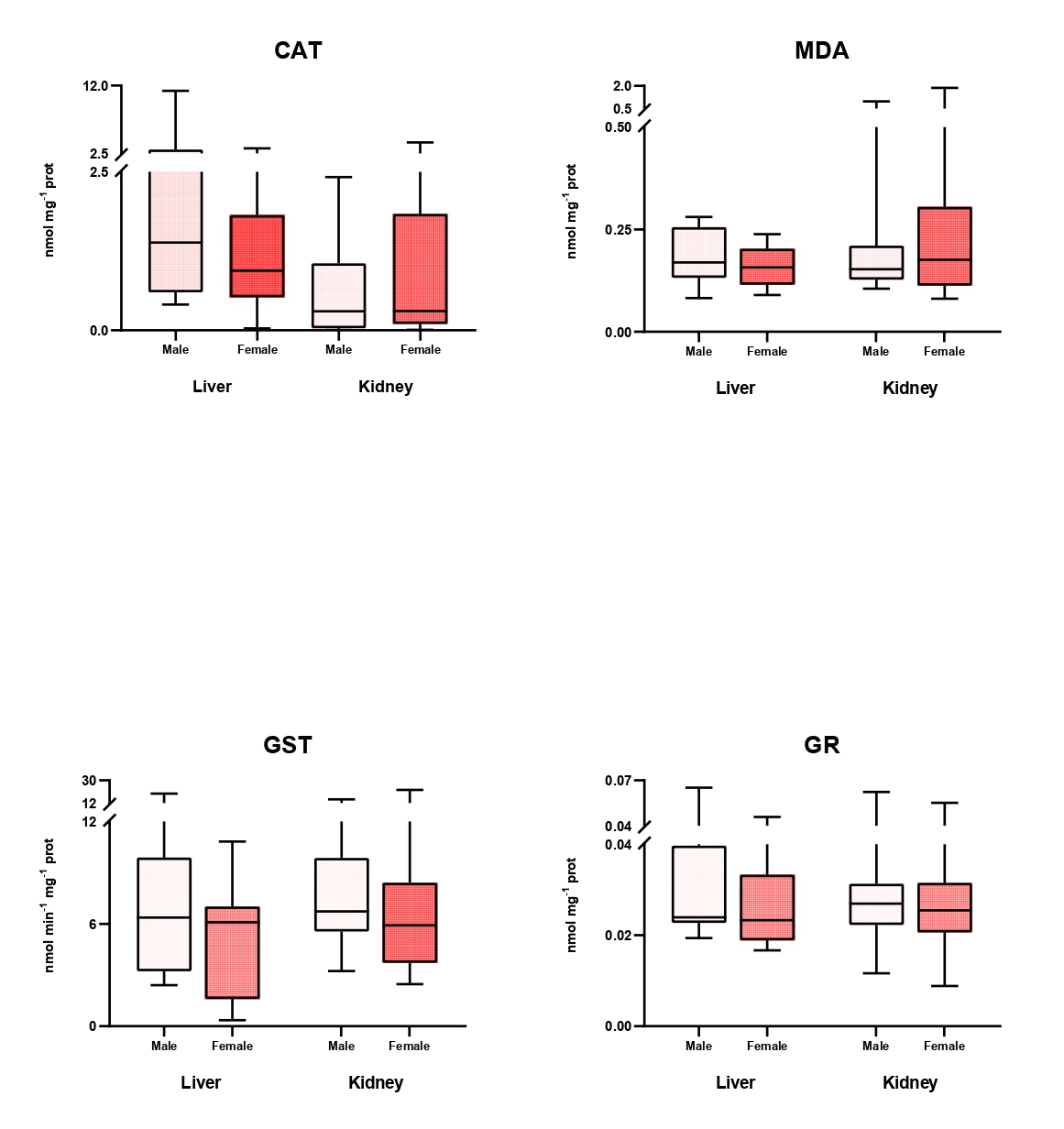


**Fig. S2**. CAT, MDA, GST and GR levels in liver and kidney of European otter according to sex. Box plots represent median values and 25-75 percentiles.
